# Development and identification of three functional markers associated with starch content in lotus (*Nelumbo nucifera*)

**DOI:** 10.1038/s41598-020-60736-6

**Published:** 2020-03-06

**Authors:** Teng Cheng, Xingwen Zheng, Keqiang Xie, Jiangdong Liu, Xingfei Zheng, Surong Jin, Ying Diao, Zhongli Hu, Jianxiong Wang

**Affiliations:** 1 State Key Laboratory of Hybrid Rice, Hubei Lotus Engineering Center, College of Life Sciences, Wuhan University, Wuhan, 430072 P. R. China; 2 Guangchang Bailian Institute of Jiangxi Province, Guangchang, 344900 P. R. China; 3 College of Life Sciences, Wuhan University, Wuhan, 430072 P. R. China; 4 School of Chemistry, Chemical Engineering and Life Science, Wuhan University of Technology, Wuhan, 430070 P. R. China; 5 College of Forestry and Life Sciences, Chongqing University of Arts and Sciences, Yongchuan, 402160 China

**Keywords:** Plant breeding, Plant molecular biology

## Abstract

It have been significantly demonstrated that Hexokinase (HXK), Granule-bound starch synthase (GBSS) and ADP-glucose pyrophosphorylase (AGPase) are three critical enzymes in the starch biosynthetic pathway and are related to starch (amylose, amylopectin and total starch) content in lotus. It is important to develop functional markers in marker-assisted selection of lotus breeding. So far there have been few reports about lotus functional markers. In this study, based on insertion-deletions (INDELs) and single-nucleotide polymorphisms (SNPs), we developed three functional markers, FMHXK-E1, FMGBSS-I8 and FMAGPL-I1. FMHXK-E1 was developed based on polymorphisms of two haplotypes of *NnHXK*. 26 lotus cultivars that the 320-bp fragment presented in *NnHXK* had a lower content of amylose and a higher content of amylopectin. FMGBSS-I8 was developed based on polymorphisms of two haplotypes of *NnGBSS*. The group containing 32 lotus cultivars with the 210-bp fragment had less amylose content and more amylopectin content. FMAGPL-I1 was developed based on polymorphisms of two haplotypes of *NnAGPL* (ADP-glucose pyrophosphorylase large subunit gene). The group containing 40 lotus cultivars with the 362-bp fragment had less amylopectin, total starch content and more amylose content. According to the study, FMHXK-E1, FMGBSS-I8 and FMAGPL-I1 are closely related to lotus starch content. It could be provided research basis for molecular assisted selection of lotus starch content improve breeding efficiency.

## Introduction

Lotus (*Nelumbo nucifera* Gaertn), a perennial aquatic herb, is one of the oldest dicotyledonous plants^1^, which originated and has been widely grown in southern China for thousands of years^2^. *Nelumbo* Adans, a surviving living fossil that has experienced the Quaternary glacial period, has an evolutionary history of almost 135 million years. In addition to the evolution values, lotus is also a kind of essential traditional Chinese medicine and food and its rhizome has been widely consumed for over 7000 years in Asia^3^. Starch content is one of the main factors affecting the lotus root processing and cooking quality. Starch can be divided into amylose and amylopectin in plant^4^. Amylose is a 200 glucose groups polysaccharide linear molecule with glucose residues linked together by α-(1,4) glycosidic bonds. Amylopectin is a 300 to 400 glucose groups chain molecule linked together by α- (1,4) glycosidic bonds and α- (1,6) glycosidic bonds^5,6^.

Identifying genes and molecular markers associated with trait variation is obligatory for comprehending molecular breeding and crop improvement^7^. Up to now it is possible to develop markers from genes that have a putative function which is referred to as ‘functional markers’ (FMs)^8^. FMs that developed from gene polymorphisms affect phenotypic trait variation^8,9^. Therefore, it is necessary to understand the function of genes in the development of functional markers. In recent years, the new type of molecular marker (FM), based on the insertion/ deletion (INDEL) and the single-nucleotide polymorphism (SNP), has been successfully developed and play a broader role in plant molecular marker-assisted breeding^10–12^. For example, the functional marker of *GBSS* has been used to select wheat materials, which is linked with flour quality^13^. Although traditional SSR and ISSR analysis of genetic diversity have been used in lotus cultivars^14–16^, functional markers associated with starch content have not been developed and applied in lotus cultivars. So, it is a critical step towards selecting suitable lotus cultivars to develop functional markers on the identification of amylose, amylopectin and total starch content.

Starch is only produced through biosynthetic pathway, which involves lots of conservative function enzymes, such as hexokinase (HXK), granule-bound starch synthase (GBSS), ADP-glucose phosphorylase (AGP), soluble starch synthases (SSS), starch branching enzymes (SBE), starch debranching enzymes (DBE) and so on^4,17^. Numerous studies showed that granule-bound starch synthase was encoded by the GBSS gene, which catalyzes amylose synthesis^18–20^. AGP is a rate-limiting enzyme which catalyzes ATP and Glc-1-P to pyrophosphate and ADP-glucose (ADPG). ADPG acts as the substrate for the synthesis of amylose and amylopectin under the action of others starch synthases^21–23^. HXK could provide a carbon stream for plant starch synthesis which catalyzes fructose to Glc-1-P^6^. Identifying genes that control starch content could contribute to explore molecular markers about starch content.

In this study, 10 genes, hexokinase gene (*HXK*), granule-bound starch synthase gene (*GBSS*), ADP-glucose phosphorylase gene (*AGP*), soluble starch synthases gene (*SSS*), starch branching enzymes gene (*SBE*), sucrose synthase gene (*SUSY*), fructokinase gene (*FRK*), UDP glucose pyrophosphrylase gene (*UGP*), isoamylase gene (*ISA*) and pullulanase gene (*PUL*), were employed to develop functional markers. Our group have resequenced to >13× raw data of 69 lotus accessions (accession no. SRP095218). On the basis of these genomic data and the starch biosynthetic pathway, the SNP and Indel were identified in lotus. We validated the Indel and SNP in 46 different cultivars and further analysis revealed that three enzymes, HXK, GBSS and AGPL, significantly associated with starch content are encoded by Indel or SNP of genes. Finally, we respectively developed three functional markers, FMHXK-E1, FMGBSS-I8 and FMAGPL-I1, from *HXK*, *GBSS* and *AGP*L.

## Results

### Development and identification of FMHXK-E1

**Figure 1:**
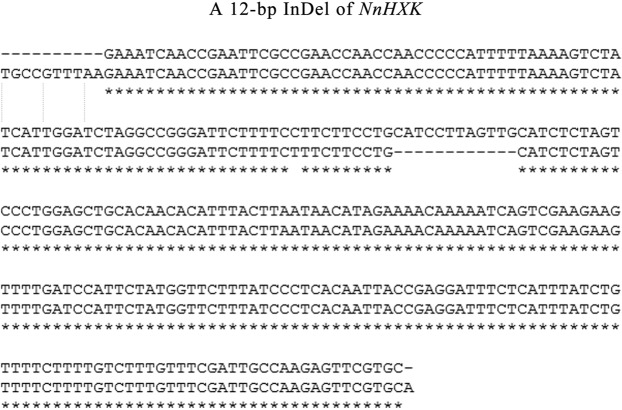
Indel of *NnHXK*. The 12-bp InDel region in first exon of *NnHXK* is indicated in the white background.

The 320 bp and 308 bp fragment sequences of *NnHXK* were detected by primer HXK-1E (Table 1). An inserted/deleted fragment of 12-bp was found in the exon of *NnHXK* by blasting result of PCR product sequencing (Fig. 1). Based on the results of PCR detection, a pair of alleles with 308-bp and 320-bp fragment were detected (Supplementary File A). In order to investigate the effect of the 12-bp Indel on starch content, 320-bp fragment differences in 46 lotus accessions were analyzed. It was presented by Excel analysis that the amylose content is lower and the amylopectin content is higher of 26 lotus cultivars with the 320-bp fragment of *NnHXK*, and the amylose content is higher and the amylopectin content is lower of the another 20 lotus cultivars without the 320-bp fragment of *NnHXK*. The percentage of amylose in dry matter (5.23%) and the percentage in total starch (22.09%) are both significantly higher than the percentage of amylopectin in dry matter (3.13%) and the percentage in total starch (11.53%). Correlation analysis showed that the significance differences of amylose and amylopectin content in total starch with diversity bands of marker reached a high level. In FMHXK-E1, with diversity bands of marker, correlation analysis showed significant differences in amylose and amylopectin content in total starch that P value reached a high level at 0.010 and 0.008(P ≦ 0.01) respectively. P value reached a very level at 0.007 (P ≦ 0.01) of amylose content in dry matter (Table 2). The functional marker was developed and named FMHXK-E1 according to the gene HXK and primer.

**Table 1:** Locus and Primer information.

Locus (accession Number or reference)	Encoded protein	Primer name	Primer sequence (5′-3′) or reference	PCR product size (bp)	Annealing temperature (°C)
The first exon of *HXK*	Hexokinase	HXK-E1	f-tct aaa tcc caa tcc gtc c	308 + 320	51
			r-gca cga act ctt ggc aat c		
The eighth intron of *GBSS*	Granule-bound starch synthase	GBSS-I8	f-ggc att act ggt att gtg a	210 + 220	53
			r-gct tcc ttt aga aga ggc t		
The first intron of *AGPL*	ADP-glucose phosphorylase large subunit	AGPL-I1	f-tgg att ctt gtt gtg cga c	0 + 362	57
			r1-tgg aaa gaa tag cct ggg		
			r2-tgg aaa gaa tag cct gtg

**Table 2:** The results of t-test FM*HXK*-E1.

No.	item	PCR products without 320 bp fragment	PCR products with 320 bp fragment	t value	p value	significance level
1	the percentage of amylose in dry matter, %	5.23	3.13	2.83	0.007	**
2	the percentage of amylopectin in total starch, %	77.89	88.08	−2.67	0.010	**
3	the percentage of amylose in total starch, %	22.09	11.53	2.76	0.008	**
4	the percentage of amylopectin in dry matter, %	23.94	30.70	−1.59	0.119	NS
5	the percentage of total starch in dry matter, %	29.18	33.83	−1.16	0.254	NS

**Indicated that the difference reached a very significant level, *Indicated that the difference reached a significant level, “NS” indicated that the difference was not significant.

### Development and identification of FMGBSS-I8

**Figure 2:**
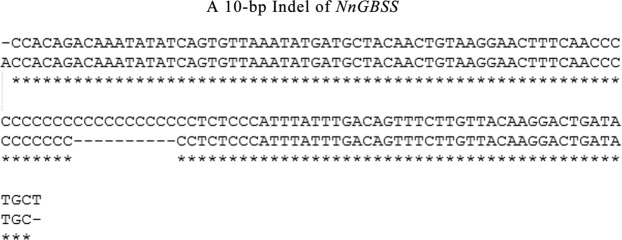
Indel of *NnGBSS*. There is a 10-bp InDel in the eighth intron of Nn*GBSS II-1*. The 10-bp InDel region in the eighth intro of *NnGBSS II-1* is indicated in white background.

The 210-bp and 220-bp fragment sequences of *NnGBSS* were detected by primer GBSS-8I (Table 1). An inserted/deleted fragment of 10-bp was found in the intron of *NnGBSS* by blasting result of PCR product sequencing (Fig. 2). A pair of alleles with 210-bp and 220-bp fragment were detected according to the results of PCR detection (Supplementary File B). In order to explore the influence of the 10-bp Indel on starch content, 210-bp fragment differences in 46 lotus cultivars were analyzed. According to the result of t-test analysis, it was showed that 32 lotus cultivars with the 210 bp fragment of *NnGBSS* had lower amylose content and higher amylopectin content and another 14 lotus cultivars missing the 210-bp fragment of NnGBSS contained more amylose and less amylopectin. The amylose percentage in dry matter (5.26%) and in total starch (23.56%) of 14 lotus cultivars missing the 210-bp fragment are both higher than the amylopectin percentage in dry matter (3.51%) and in total starch (13.09%) of 32 lotus cultivars with the 210 bp fragment. Apparently, there was a significant correlation between the PCR bands diversity and starch (amylose and amylopectin) content. In FMGBSS II−1, with diversity bands of marker, correlation analysis showed significant differences in amylose and amylopectin content in total starch that P value at 0.040 and 0.022(P ≦ 0.05) respectively. The P value at 0.026 (P ≦ 0.05) of amylose content in dry matter (Table 3). The functional marker was developed and named FMGBSS-I8 according to the gene GBSS and primer.

**Table 3:** The results of t-test FM*GBSS*-I8.

No.	item	PCR products without 210 bp fragment	PCR products with 210 bp fragment	t value	p value	significance level
1	the percentage of amylose in dry matter, %	5.26	3.51	−2.11	0.040	*
2	the percentage of amylopectin in total starch, %	76.94	86.59	2.31	0.026	*
3	the percentage of amylose in total starch, %	23.56	13.09	−2.37	0.022	*
4	the percentage of amylopectin in dry matter, %	22.26	30.17	1.74	0.089	NS
5	the percentage of total starch in dry matter, %	27.41	33.54	1.43	0.171	NS

*Indicated that the  difference reached a significant level, NS indicated that the difference was not significant.

### Development and identification of FMAGPL-I1

**Figure 3:**
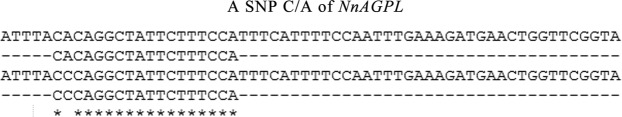
SNP of *NnAGPL*. A SNP C/A in the first intron of Nn*AGPL*. The second and fourth lines are complementary sequences of r-2 and r-1, respectively.

A SNP of C/A sequences of *NnAGPL* was found in the first intron of *NnAGPL* by primer AGPLI1 (Fig. 3) (Table 1). According to the principle of ARMS (The amplification refractory mutation system)^24–27^, AGPLI1 had the same forward primer, and the second bases of 3′-reverse primers were complementary each other. Through forward primer-f and reverse primer-r1, PCR amplification yielded a 362-bp fragment in 40 lotus cultivars. The other 6 lotus cultivars lacked the 362-bp fragment, because the mutant carried a point mutation in the intron of *NnAGPL*, where base corresponded to the second base of 3′-reverse primer (Supplementary File C). In order to study the effect of the mutant on starch content, 210-bp fragment differences in 46 lotus cultivars were analyzed. The total starch content in the lotus with SNP site at C base was lower (total starch accounted for 30.2% in dry matter), while the total starch content in the lotus with SNP site at A base was higher (total starch accounted for 42.23% in dry matter) According to the analysis of t-test, the results showed that the polymorphism of *NnAGPL* was significantly correlated with the starch content (Table 4). Association analysis showed that total starch had a significant difference (P = 0.042) with fragment polymorphism (P ≤ 0.05). FMAGPL-I1 could directly screen out lotus varieties with high or low total starch content. The functional marker was developed and named FMAGPL-I1 according to the gene AGPL and primer name.

**Table 4:** The results of t-test FM*AGPL*-I1.

No.	item	PCR products without 362 bp fragment	PCR products with 362 bp fragment	t value	p value	significance level
1	the percentage of amylose in dry matter, %	25.90	40.20	2.36	0.023	*
2	the percentage of amylopectin in total starch, %	4.35	2.04	−2.03	0.048	*
3	the percentage of amylose in total starch, %	30.24	42.23	2.09	0.042	*
4	the percentage of amylopectin in dry matter, %	82.05	94.30	2.12	0.039	*
5	the percentage of total starch in dry matter, %	17.68	5.71	−2.06	0.045	*

*Indicated that the difference reached a significant level.

## Discussion

Functional markers is a novel DNA molecular markers, which were developed from polymorphic motifs of functional genes causing differences in phenotypic traits. The association analysis of plant population phenotypic characteristics and functional genes of phenotypic correlation is a method to develop indirect functional markers. With the separation and annotation of functional genes, functional markers are gradually becoming a new type of DNA molecular markers since random DNA molecular markers, which can greatly improve the efficiency and accuracy of labeling. Compared with the other DNA molecular markers, functional markers have a broad application prospect in assisted genetic breeding and variety identification, because they are completely related to polymorphism sequences of functional genes. Iyer-pascuzzi and McCouch developed functional markers from functional single nucleotide polymorphisms of *xa-5*, the gene of rice blast disease resistant, which can quickly and accurately select the rice varieties with resistance to rice blast disease and greatly improve the breeding process^28^.

There was the explanation allelic variation at some loci might indeed be causal for the trait variation for this finding. And INDELs or SNPs polymorphisms were performed to significantly affect characters of agronomic^29^. The possible molecular mechanism was that amino acid changes had contributed to the variation of enzyme activity, stability or post-translational modifications, which altered protein conformation or modification sites, or the variation in expression level due to DNA polymorphisms in cis-regulatory sequences. In previous studies on wheat phytoene synthase 1 gene (*Psy1*), a 37-bp insertion in the second intron exhibited significant association with wheat *Psy1* (phytoene synthase 1) activity^30^. Similar study had certificated SNP A/G polymorphisms significant associated with Dhn1 and Rsp41 activity for the drought resistance^10^.

At present, conventional breeding methods are inefficient in improving the starch traits of lotus roots. For this case, functional markers developed from genes that control enzymes involved in starch, is more wisely to select plants in the seedling stage. It can accelerate genetic breeding process by improving the lotus root quality and traits. The experiment of this study is an association tests for starch content traits in advanced cultivars and breeding materials of lotus root. Three functional markers, FMHXK-E1, FMGBSS-I8 and FMAGPL-I1, were developed based on sequence polymorphism among genotypes. In FMHXK-E1 and FMGBSS-I8 markers, amylose and the amylopectin content in the dry matter and total starch have significant differences with fragment polymorphism, but they are different in degree. The difference between FMHXK-E1 and starch content is extremely significant (P≤0.01), FMGBSS-I8 and starch content are significantly different (P≤0.05). In FMAGPL, total starch in dry matter had a significant difference (P=0.042) with fragment polymorphism. All of the loci were selected based on co-localization of functional candidate genes for starch content^31–33^. Target lotus root with suitable starch content could be selected from different lotus root cultivars and our results illustrated these functional markers were credible as reliable markers for molecular assisted selection of lotus starch content. The markers are closely linked to the target genes. Compared to the traditional way that measuring the starch content of rhizomes in the later stage of lotus growth, it provides the possibility for selection in the early stage and single plant can be selected as the test object. It can greatly reduce the blindness of the breeding process, shorten the breeding life and improve breeding efficiency.

## Materials and Methods

### Test materials and genomic DNA extration

46 different cultivars of lotus roots were provided by the Guangchang Bailian Institute of Jiangxi Province, China (Table 5). Genomic DNA was extracted from the leaf tissue of 46 cultivars by plant genomic DNA kit (TIANGEN Co. LTD) in accordance with the manufacturer’s instructions and ran on 1% agarose gel for quality evaluation.

**Table 5:** The information of lotus samples and starch content used in this study.

No.	Name	Sources	the percentage of amylopectin in dry matter, %	the percentage of amylose in dry matter, %	the percentage of total starch in dry matter, %	the percentage of amylopectin in total starch, %	the percentage of amylose in total starch, %
1	Thailand lotus-I	Thailand	20.4	2.9	23.3	87.55	12.45
2	Cunsan lotus	Xiangtan, Hunan	14.63	4.48	19.11	76.54	23.45
3	Baixiang lotus	Xiangtan, Hunan	16.71	3.78	20.49	81.55	18.46
4	Hongxiang lotus	Xiangtan, Hunan	5.38	2.63	8.01	67.18	32.82
5	Dongtinghu wild lotus	Dongtinghu, Hunan	51.01	6.02	57.03	89.43	10.57
6	Nangeng wild lotus	Ma’anshan, Anhui	18.08	10.95	29.03	62.27	37.73
7	Tuxuan lotus	Jinhua, Zhejiang	28.43	5.26	33.69	84.38	15.32
8	Chuzhoubai lotus	Jinhua, Zhejiang	13.12	9.09	22.21	59.08	40.92
9	Baihuajian lotus	Jinhua, Zhejiang	34.39	3.53	37.92	90.69	9.31
10	Honghuajian lotus	Jinhua, Zhejiang	22.54	6.13	28.67	78.63	21.37
11	Baiye lotus	Guangchang, Jiangxi	30.87	4.56	35.43	87.13	12.87
12	Jingguang-2 hao	Guangchang, Jiangxi	9.09	3.89	12.98	70.01	29.91
13	Xingkongmudan	Guangchang, Jiangxi	14.71	2.86	17.57	83.71	16.29
14	Gudai lotus	Wuhan, Hubei	45.41	2.29	47.7	95.19	4.81
15	Zhongnanhaigu lotus	Wuhan, Hubei	60.86	3.53	64.39	94.51	5.49
16	Thailand lotus-VI	Thailand	25.99	2.21	28.2	92.15	7.85
17	Puzheheibai lotus	Wuhan, Hubei	29.29	3	32.29	90.70	9.3
18	Zhanhongtu	Beijing	27.13	8.16	35.29	76.87	23.13
19	Baiyangdianhong lotus	Nanjing, Jiangsu	36.74	1.02	37.76	97.31	2.69
20	Zhuanshang lotus	Beijing	34.9	8.43	43.33	80.55	19.45
21	Jiandehonghua lotus	Jiande, Zhejiang	12.94	9.67	22.61	57.24	42.76
22	Sumudan	Guangchang, Jiangxi	34.23	1.01	35.24	97.14	2.86
23	Taikong 36 hao	Guangchang, Jiangxi	33	1.9	34.9	94.54	5.46
24	Jingguang-2 hao	Guangchang, Jiangxi	25.47	2.88	28.35	89.82	10.18
25	Jianxuan-17 hao	Jianning, Fujian	44.75	1.52	46.27	96.70	3.3
26	Thailand lotus-III	Thailand	14.84	2.93	17.77	83.52	16.48
27	Thailand lotus-IV	Thailand	8.81	8. 74	17.55	50.19	49.81
28	Thailand lotus-V	Thailand	46.37	1.79	48.16	96.29	3.71
29	Jianxuan-35 hao	Jianning, Fujian	6.87	10.53	17.4	39.48	60.52
30	Jinfurong-2 hao	Jinhua, Zhejiang	15.99	6.35	22.34	71.56	28.44
31	Jianxuan-30 hao	Jianning, Fujian	7.6	3.58	11.18	67.98	32.02
32	Changezuiwu	Guangchang, Jiangxi	33.39	1.8	35.19	94.88	5.12
33	Taikongjiaorong	Guangchang, Jiangxi	46.01	3.7	49.71	92.56	7.44
34	Fengjuanhongqi	Guangchang, Jiangxi	24.32	3.25	27.57	88.19	11.81
35	Cuihehongyan	Guangchang, Jiangxi	16.78	3.08	19.86	84.47	5.53
36	Lvguozijuan	Guangchang, Jiangxi	23.97	4.2	28.17	85.09	14.91
37	Baihu wild lotus	Anhui	22.37	5.79	28.16	79.45	20.55
38	Shanmiao wild lotus	Anhui	15.55	2	17.55	88.62	11.39
39	Gan-62 hao	Guangchang, Jiangxi	31.04	3.58	34.62	89.65	10.35
40	Donggua lotus	Changsha, Hunan	36.41	1.14	37.55	96.95	3.05
41	Qingtang wild lotus	Anhui	53.08	1.84	54.92	96.64	3.36
42	Diaochahu lotus	Hanchuan, Hubei	56.01	1.54	57.55	97.32	2.68
43	Yixian lotus	Nanjing, Jiangsu	16.79	3.99	20.78	80.79	19.21
44	Wuxi lotus	Wuxi, Jiangsu	48.42	1.29	49.71	97.40	2.6
45	Furong lotus	Xiangtan, Hunan	16.71	2.2	18.91	88.36	11.64
46	Lianhu wild lotus	Anhui	45.68	1.08	45.76	97.69	2.31

### Determination of starch content in lotus root

Amylose, amylopectin and total starch content were measured both following a protocol of Williams V R *et al*.^34^ based on at least three main roots selected from 46 lotus roots. Then the proportion of each component were calculated according to amylose content and amylopectin content. (Table 4).

### Primer design and synthesis

The whole genome sequence of Chinese Lotus had already been made public^35^. Genomic DNA sequence of *NnHXK* was also obtained Gene ID:104592043. The NnGBSS gene had been cloned, and genomic DNA and cDNA sequence of *NnGBSS* were deposited to the GeneBank (GenBank accession no. FJ602702)^18,36^. Full length cDNA sequence of *NnAGPS1* (GeneBank accession no. KJ476823) and *NnAGPL* (GeneBank accession no.KJ476824) were isolated and deposited to the GeneBank. Genomic DNA sequence of *NnAGPS1* (GeneBank accession no. KJ476825) and *NnAGPL* (GeneBank accession no. KJ476826) were also obtained and sequenced^21^.

According to Genomic DNA and cDNA sequences of genes, primers were designed by using the software Primer Premier 5.0 meeting the following constraints: GC content of 40–60%, 18–22 nucleotides in length, no secondary structure, and no consecutive tracts of a single nucleotide. All primers were synthesized by AuGCT Corporation (Beijing, Co. LTD).

### PCR amplification and PAGE electrophoresis

The total volume of 25 μL mixture containing 1 μL *Taq* DNA polymerase, 1.4 μL genomic DNA, 1.5 μL forward primer (20 μM), 1.5 μL reverse primer (20 μM), 2.5 μL of 10× buffer, 0.6 μL dNTPs (5 mM each), and 15.5 μL ddH_2_O. The PCR cycling was performed in a LifePro Thermal Cycler and amplifyed under the following conditions: 94 °C for 5 min, 35 cycles of 30 s at 94 °C, 30 s at 51–63 °C, 45 s at 72 °C, and a final extension for 5 min at 72 °C.

The products of PCR amplified were mixed with loading buffer, denatured for 6 min at 95 °C, and utilized with a size standard marker of pBR322 DNA/Msp 1 (TIANGEN) to each lane. The products were analyzed by polyacrylamide gel (PAGE) electrophoresis on 6% acrylamide, visualized by silver ion staining and photographed.

### Association analysis

Based on the p/a(present/absent) of DNA bands (certain allele), all materials could be divided into two groups. The number of samples from each group were analysis by Excel double sample equal variance t-test method. Based on the p/a amplified bands, we divided respectively amylose, amylopectin and total starch content into two groups. Two groups data were performed for correlation analysis.

## Supporting Information

Supporting Information.
