# Supplementary material for: Development and identification of three functional markers associated with starch content in lotus (Nelumbo nucifera)
Source: Sci Rep. 2020 Mar 6;10:4242. doi: 10.1038/s41598-020-60736-6 (PMC7060276; doi:10.1038/s41598-020-60736-6)
Supplement: Supplementary file 1 — Supporting Information. [file 41598_2020_60736_MOESM1_ESM.pdf]

Development and identification of three functional markers associated with starch content in lotus (*Nelumbo nucifera*)

Teng Cheng[1], Xingwen Zheng[1], Keqiang Xie[2], Jiangdong Liu[3], Xingfei Zheng[1], Surong Jin[4], Ying Diao[1,5], Zhongli Hu[1]*, Jianxiong Wang[1]*

[1]State Key Laboratory of Hybrid Rice, Hubei Lotus Engineering Center, College of Life Sciences, Wuhan University, Wuhan, 430072, P.R. China

[2]Guangchang Bailian Institute of Jiangxi Province, Guangchang, 344900, P.R. China

[3]College of Life Sciences, Wuhan University, Wuhan, 430072, P.R.China;

[4]School of Chemistry, Chemical Engineering and Life Science, Wuhan University of Technology, Wuhan, 430070, P.R.China

[5]College of Forestry and Life Sciences, Chongqing University of Arts and Sciences, Yongchuan 402160, China

* Corresponding author: Zhongli Hu (email: huzhongli@whu.edu.cn) & Jianxiong Wang (email: 214065995@qq.com)

Supplementary file Polymorphism of primer HXK-E1, GBSS-I8, AGPL-I1. A) Genotypes of 46 kinds of lotus roots could clearly discriminated by HXK1E according to band size and pattern. B) Genotypes of 46 kinds of lotus roots could clearly discriminated by GBSS8I according to band size and pattern. C)Genotypes of 46 kinds of lotus roots could clearly discriminated according to band pattern.

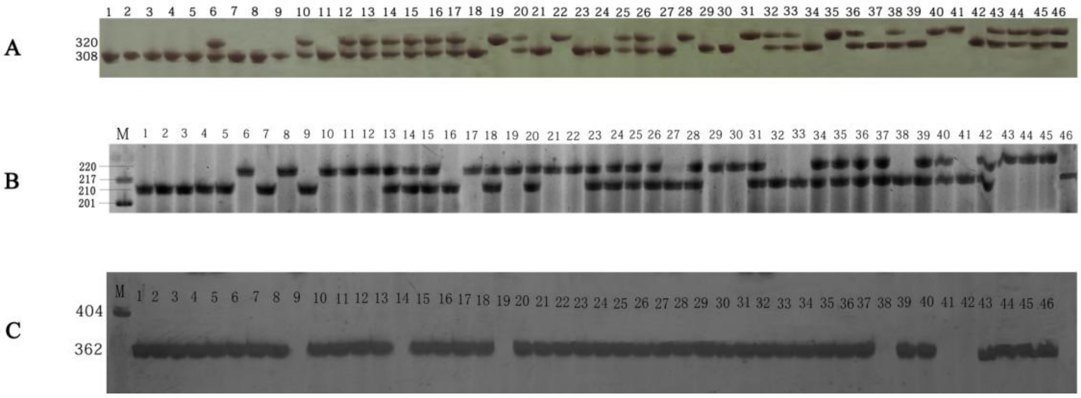
A
320
308
B
M
220
217
210
201
C
M
404
362
